# The interactions of molecular chaperones with client proteins: why are they so weak?

**DOI:** 10.1016/j.jbc.2021.101282

**Published:** 2021-10-06

**Authors:** Taylor Arhar, Arielle Shkedi, Cory M. Nadel, Jason E. Gestwicki

**Affiliations:** Department of Pharmaceutical Chemistry and the Institute for Neurodegenerative Disease, University of California San Francisco, San Francisco California, USA

**Keywords:** chaperone, protein folding, protein aggregation, protein–protein interactions (PPIs), nuclear magnetic resonance (NMR), Hsp27, heat shock protein 27, JDP, J-domain protein, NBD, nucleotide-binding domain, NMR, nuclear magnetic resonance, PPD, peptidyl-prolyl-cis/trans isomerase domain, PPI, protein–protein interaction, PTM, posttranslational modification, RBD, ribosome-binding domain, RNC, ribosome-nascent-chain, SBD, substrate-binding domain, sHsp, small heat shock protein, TF, trigger factor

## Abstract

The major classes of molecular chaperones have highly variable sequences, sizes, and shapes, yet they all bind to unfolded proteins, limit their aggregation, and assist in their folding. Despite the central importance of this process to protein homeostasis, it has not been clear exactly how chaperones guide this process or whether the diverse families of chaperones use similar mechanisms. For the first time, recent advances in NMR spectroscopy have enabled detailed studies of how unfolded, “client” proteins interact with both ATP-dependent and ATP-independent classes of chaperones. Here, we review examples from four distinct chaperones, Spy, Trigger Factor, DnaK, and HscA-HscB, highlighting the similarities and differences between their mechanisms. One striking similarity is that the chaperones all bind weakly to their clients, such that the chaperone–client interactions are readily outcompeted by stronger, intra- and intermolecular contacts in the folded state. Thus, the relatively weak affinity of these interactions seems to provide directionality to the folding process. However, there are also key differences, especially in the details of how the chaperones release clients and how ATP cycling impacts that process. For example, Spy releases clients in a largely folded state, while clients seem to be unfolded upon release from Trigger Factor or DnaK. Together, these studies are beginning to uncover the similarities and differences in how chaperones use weak interactions to guide protein folding.

The complex problem of protein folding has fascinated generations of scientists, as it has implications for a wide range of fields, including protein engineering, protein therapeutics, and the study of protein misfolding diseases. Pioneering work by Anfinsen demonstrated that a protein's primary sequence typically contains the information needed to adopt the native state (1). The forces that stabilize this native structure come primarily from favorable hydrophobic contacts, along with additional contributions from polar interactions (*e.g.*, cation-π, H-bonds, etc.) (2, 3). However, in the crowded environment of the cell, high concentrations of biomolecules, along with other constraints, create nonideal folding conditions. In this scenario, off-pathway processes, such as aggregation or misfolding, can become significant contributors. These off-pathway events can reduce the level of the folded, functional protein, while also leading to accumulation of proteotoxic structures. To protect against these possibilities, the cell relies on a large class of dedicated proteins, the molecular chaperones, to monitor the folding of the proteome.

There are 100+ genes for molecular chaperones in the human genome. The resulting chaperone proteins vary considerably in their sequences, sizes, and shapes (Fig. 1*A*). Some chaperones, such as Spy of the bacterial periplasm and heat shock protein 27 (Hsp27) of the eukaryotic cytosol, are relatively small (<30 kDa) and lack any domains with enzymatic activity. Others, such as Hsp70 and Hsp90, are larger and have the ability to hydrolyze ATP. Some chaperones, such as Tric/CCT and GroEL, form stable, high molecular mass, barrel-shaped, structures. At the sequence level, there is no conservation among the different classes of chaperones; there is no “chaperone fold.” Rather, the categories of chaperones are wildly divergent in size and shape (Fig. 1*A*). Yet, despite these differences, the chaperones share a common function: they promote the folding of other proteins. For example, a commonly used hallmark of chaperone function is that they will promote the refolding of denatured proteins *in vitro* (4). In that process, the chaperone limits aggregation of the denatured protein and promotes restoration of the native state. Remarkably, this activity is not restricted to any particular structural class of chaperone, nor is it a product of only chaperones with ATP hydrolysis activity (Fig. 1, *B* and *C*). Rather, there is something more fundamental about chaperone functions, which is not immediately apparent in their structure or sequence. Also, some chaperones are able to promote this process by themselves. However, it is also common for multiple categories of chaperones to work together, engaging in protein–protein interactions with each other and with their “client” proteins (5, 6, 7, 8, 9).

**Figure 1:**
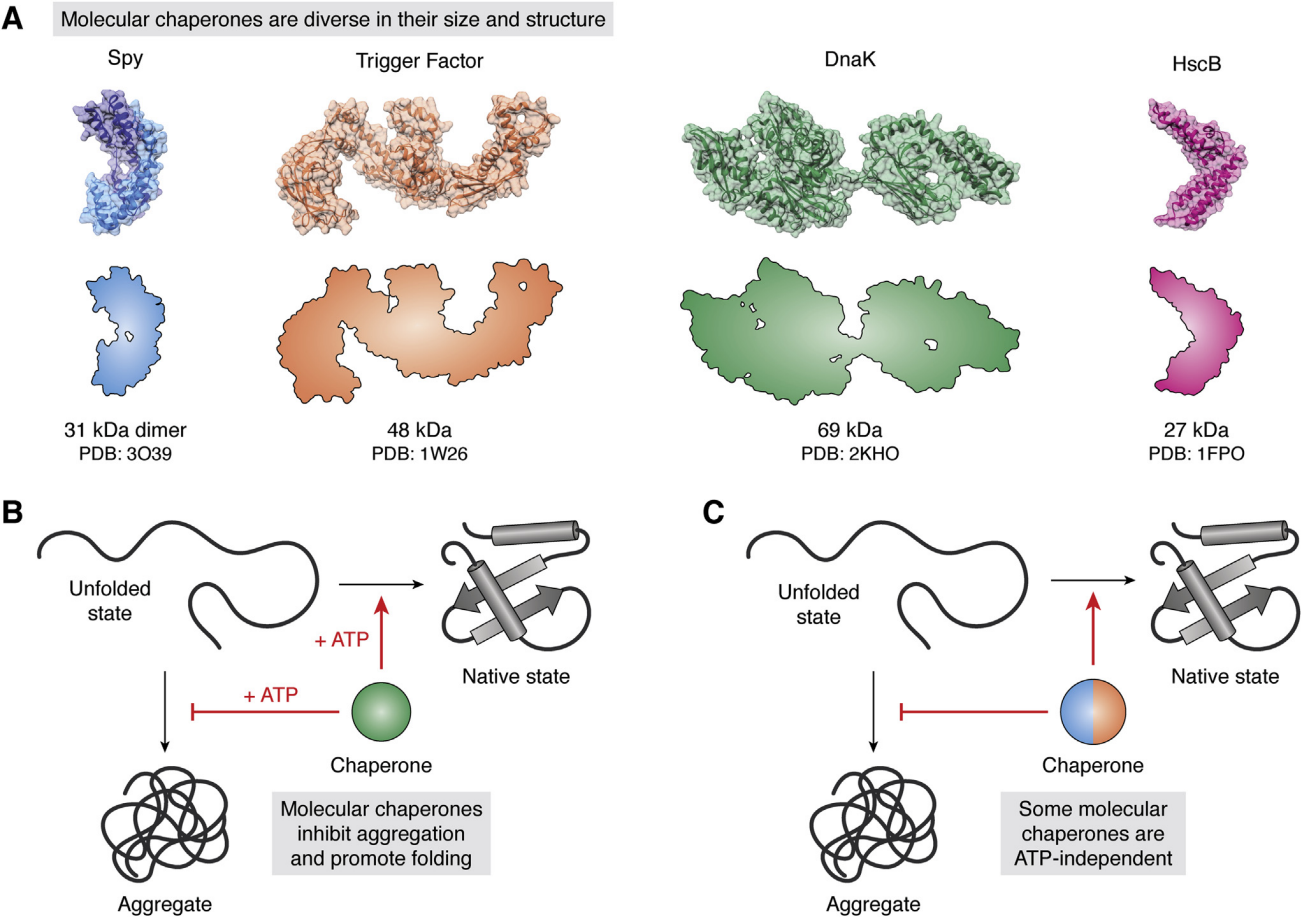
**Structurally diverse molecular chaperones direct clients to the native state.***A*, comparison of four molecular chaperones that differ in size, sequence, and shape. The molecular mass and PDB code for each chaperone are shown. Despite these different architectures, the chaperones all promote client folding and limit aggregation. *B* and *C*, core functions of the molecular chaperones are to suppress aggregation and promote client folding by directly binding to the unfolded state(s). While some chaperones (DnaK, *green*) require ATP to perform these functions (*B*), others (Spy, *blue*, and Trigger Factor, *orange*) work independently of nucleotide turnover (*C*).

How do chaperones work? There are some unifying features of how chaperones bind to their clients that may suggest the start of an answer. Most molecular chaperones bind their clients *via* weak, hydrophobic interactions. Compared with other protein–ligand or protein–protein interactions (PPIs), chaperone–client interactions are particularly weak and transient (10). Presumably, the chaperones have evolved to possess weak affinity, and this feature appears to have some benefits. For example, the relatively weak affinity and poor shape complementarity of chaperone–client interactions seem to allow the chaperone to recognize a wide range of different sequences (11, 12, 13). This aspect is likely important in allowing the limited number of chaperones to act on the entire proteome. Recent studies have suggested that this malleable surface might even assist in protein evolution (14). However, there is another potential reason for why chaperone–client PPIs are so weak. In the last few years, the interactions between chaperones and unfolded clients have been studied structurally for the first time, and these studies are suggesting that a hierarchy of weak-to-strong PPI affinities might be important in promoting folding. In this model, weak interactions between chaperones and clients are readily out-competed by the stronger, intramolecular driving forces of protein folding, such as hydrophobic collapse.

In this review, we discuss these recent structural insights, with the goal of highlighting the similarities and differences in how weak PPIs might be important for chaperone function. Many structural and biophysical technologies have been used to study this question, including crystallography (15), cryo-EM (16, 17), single molecule (18), and fluorescence energy transfer methods (19, 20). However, we focus on recent studies performed using nuclear magnetic resonance (NMR) spectroscopy to study specific chaperones: Spy, Trigger Factor, DnaK, and HscA-HscB (see Fig. 1*A*) and their interactions with clients. The intent of this focus is not to exclude other important contributions, but to compare results obtained using conceptually similar approaches. From this comparison, one fascinating conclusion is that, despite the wide diversity in the structures of the chaperones, the same biophysical principles seem to, in part, underlie a unifying chaperone mechanism. However, important differences are also apparent, which might suggest why the major classes of chaperones have been largely maintained through evolution.

## Spy: a chaperone surface that guides the folding trajectory

The simplest types of chaperones are those that are small and lack ATPase activity. In these systems, PPIs, and intramolecular contacts need to be finely tuned to promote flux toward the folded state. A number of factors determine the directionality of this flux, such as relative affinity of the chaperone for the native *versus* the unfolded state, and the strength of the intramolecular interactions that stabilize the native state. Furthermore, chaperone interactions with either the native or nonnative state(s) must be weak to allow for client release. If the chaperone binds too tightly, then folding would become unfavorable within the long-lived chaperone-bound complex. The bacterial chaperone Spy has served as a pioneering model for understanding these mechanisms.

Spy is a 16-kDa, ATP-independent molecular chaperone of the prokaryotic periplasm that exists as a dimer in solution (21, 22). Like many molecular chaperones, Spy is promiscuous and binds to a wide array of clients (23). Spy has strong antiaggregation properties and can inhibit amyloid formation *in vitro* and *in vivo* (24). Spy's client-binding site is a concave surface containing four hydrophobic regions surrounded by charged and polar residues (25). As Spy's client-binding site is flexible and amphiphilic, the client is able to sample conformational space while still bound to the chaperone. This flexibility simultaneously prevents the client from aggregating (by limiting exposure of hydrophobic patches that might engage in aberrant PPIs), while also allowing it to explore conformational space and achieve its native state. As evidence of the importance of this flexibility, mutations in Spy that increase flexibility within the client-binding site can enhance its chaperone activity (26).

**Figure 2:**
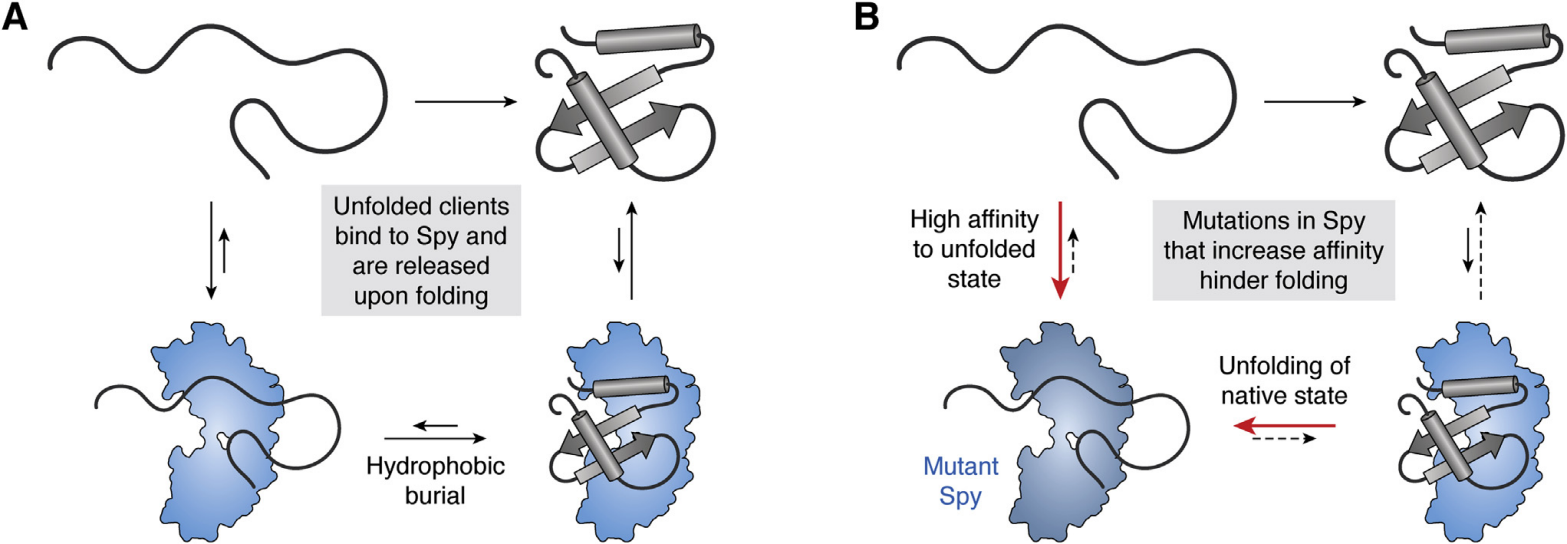
**Spy binds clients as they fold.***A*, Spy binds to unfolded clients, which may then explore diverse conformations. Upon arrival at the native state, burial of hydrophobic residues decreases Spy's affinity for the client and causes client release. *B*, When Spy's affinity for unfolded clients is increased by point mutations, this leads to unfolding of the native state and decreases the efficiency of folding.

Spy initially engages an unfolded client *via* electrostatic contacts, using the charged residues at the periphery of its client-binding site (27). This initial chaperone–client complex is subsequently stabilized by hydrophobic interactions within Spy's client-binding site. Once bound, the client becomes spatially compacted as it remains bound to Spy, reducing its conformational ensemble and generally promoting intramolecular interactions along the client folding trajectory (28). Then, folding to the native state shields the hydrophobic core of the client protein, breaking hydrophobic contacts with Spy and destabilizing the chaperone–client complex (Fig. 2*A*). While electrostatic interactions provide the initial driving force for attraction of Spy to the unfolded client, they are incapable of maintaining the chaperone–client complex following client folding (29). As such, the folded client is released from Spy. A key to this system is that Spy does not need any specific information about the folded state of the client (*e.g.*, its final structure); rather, it only provides a permissive surface on which clients might fold.

A hierarchy of interactions is critical to the mechanism by which Spy facilitates protein folding and subsequent client release. Specifically, Spy's affinity for the client decreases once it folds to its native state (K_d_ = ∼3 μM for unfolded Fyn SH3 domain *versus* K_d_ = ∼50 μM for native Fyn SH3 domain) (30). As further evidence for this model, mutations that increase hydrophobicity within the client-binding site enhance Spy's ability to prevent aggregation, but they also slow the overall rate of client folding. Moreover, these variants increase affinity for the unfolded client state to the point where their binding will unfold clients (Fig. 2*B*) (30). Such mutations are deleterious to fitness *in vivo* and are generally selected against by evolution, suggesting that this mechanism is important to Spy's function in cells. Conversely, the mutants that enhance polar contacts in the periphery of the Spy client-binding site boost antiaggregation behavior without apparent deleterious consequences to *in vivo* fitness (30). Together, these observations support the idea that electrostatic interactions are responsible for client engagement, while client release is dictated by hydrophobic collapse of the folded client. More pointedly, the interactions of Spy and client are hierarchical: the initial electrostatic contacts are replaced with hydrophobic interactions between Spy and unfolded client, which are, in turn, replaced by hydrophobic collapse of the folded client state. The rank order of these interactions allows Spy to engage its various clients while still promoting “directionality” to the natively folded state.

It has recently been demonstrated that the identity of the client itself can also greatly influence the affinity of the Spy-client PPI. Early studies of Spy–client interactions focused on small model proteins, such as Im7 and SH3, which bind Spy with low micromolar affinities (30, 31). In contrast, recent work has found that Spy binds relatively tightly (K_d_ ∼ 0.35 μM) to a nonnative state of apoflavodoxin, a topologically complex client. This high-affinity PPI is sufficient to inhibit folding; rather, Spy only binds the unfolded state and limits its aggregation (32). It is not yet clear whether this purely antiaggregation role (sometimes termed “holdase” activity) is important in the cell or whether collaboration with other “foldases” might be required for this category of Spy clients.

## Trigger factor: highly tuned interaction affinities allow a chaperone to assist in the first stages of nascent protein folding

**Figure 3:**
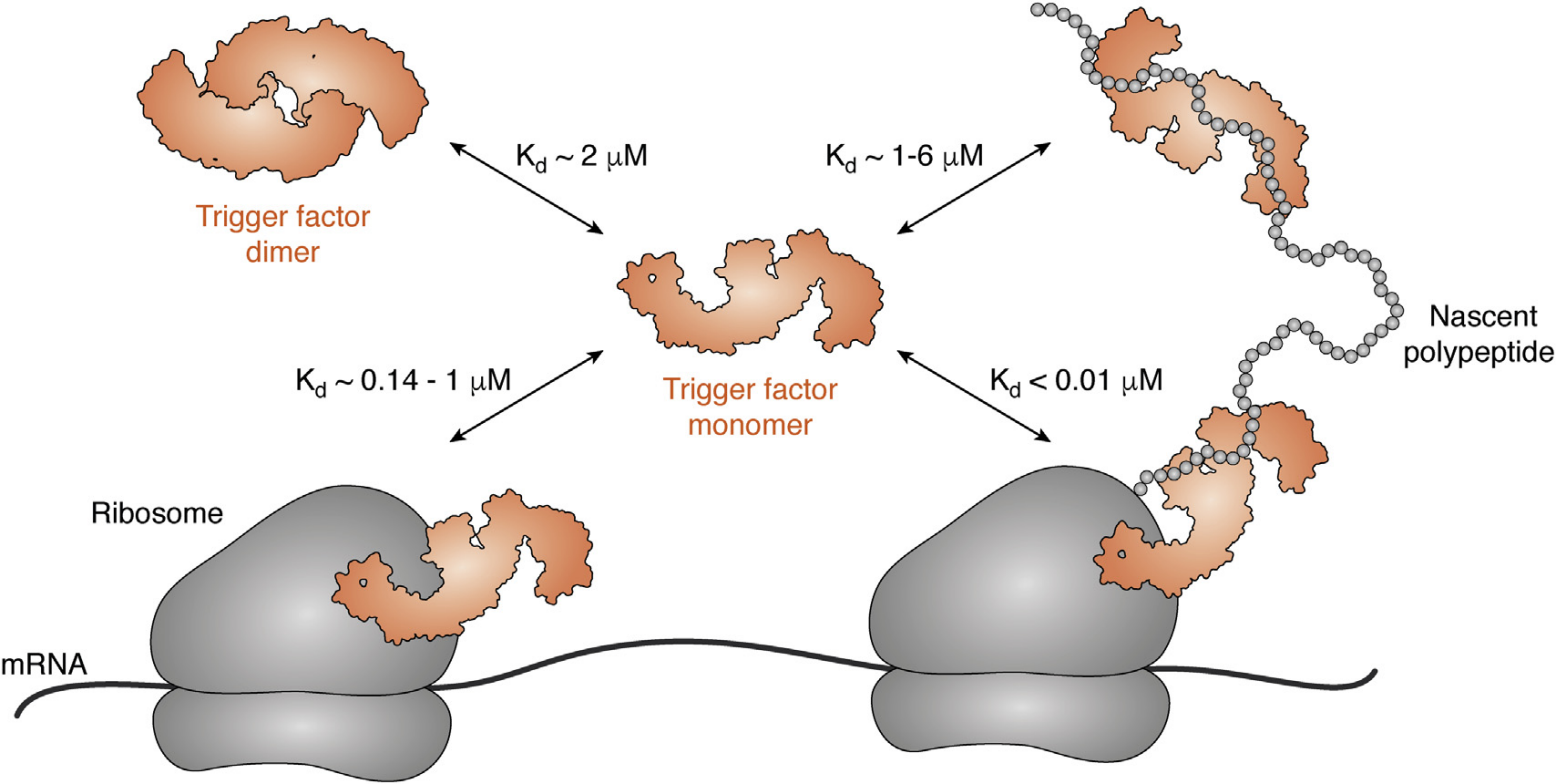
**A hierarchy of protein–protein interactions directs trigger factor (TF) to chaperone and release nascent polypeptides.** In the cytosol, TF exists in equilibrium between monomeric and dimeric states. Monomeric TF can interact with ribosomes, but its tighter affinity for translating ribosomes allows TF to selectively chaperone nascent polypeptides. As translation continues, TF can remain bound to the polypeptide and eventually dissociate to repeat the cycle.

Trigger factor (TF) is a ribosome-associated chaperone in bacteria, and it is known to assist in the earliest stages of protein folding by preventing cotranslational aggregation. TF has three major domains: a ribosome-binding domain (RBD), a substrate-binding domain (SBD), and a peptidyl-prolyl-cis/trans isomerase domain (PPD). It interacts with nascent polypeptides *via* five hydrophobic regions on its surface; four of these sites are located on the SBD, and the fifth is on the PPD (33). In solution, TF normally exists as a dimer with a head-to-tail orientation (Fig. 3) (34, 35).

Like Spy, TF is an ATP-independent chaperone. How does it direct clients to the native state? First, TF is preferentially recruited to actively translating ribosomes, known as ribosome-nascent-chain (RNC) complexes. This preference is achieved by high-affinity binding of TF to RNC complexes (K_d_ < 0.01 μM) (36, 37), which is significantly stronger affinity than its binding to the ribosome alone (reported K_d_ ranging from 0.14 to 1 μM) (34, 36, 38, 39). Importantly, TF interacts with the ribosome or the RNC as a monomer, such that the relatively tight affinity for the RNC is expected to readily outcompete the weaker dimerization constant (K_d_ ∼ 2 μM) (34, 35, 39). Together, this tight affinity and the high concentration of TF in the cytosol (∼50 μM) (40) ensure that translating ribosomes are nearly always bound by TF.

As the nascent polypeptide elongates, TF can remain bound to the nascent chain by dissociating from the ribosome (38). Thus, the process of translation and the movement of the polypeptide chain provide directionality, moving TF away from the ribosome. Compared with its tight affinity to the RNC, monomeric TF has only a modest affinity to an unfolded client (K_d_ ∼ 1–6 μM) (34, 39). This weak affinity results in competition between TF dimerization and client binding. Specifically, because TF's client-binding sites are occluded when it is dimerized, the nascent polypeptide must be released from the dimer, giving it an opportunity to engage in favorable, intramolecular interactions and begin the folding process. Another important feature of this system is that the cellular levels of TF are higher than the number of ribosome-binding sites, such that a pool of TF seems to be available to interact with the exposed client. Taken together, this hierarchy of weak PPIs (*e.g.,* with itself, with ribosome, the client, and RNC) allows TF to “find” translating ribosomes and limit cotranslational aggregation, but not bind long enough to interfere with client folding.

It seems possible that TF might use different mechanisms, depending on the nature of its client. Previous crystallographic and kinetic data has suggested that TF may also bind to small folded proteins or domains (37, 41) in a manner that is more akin to Spy's interactions described above. However, these findings contrast with recent NMR work demonstrating that small proteins interact with TF in the unfolded state (34). It seems most likely that different client proteins (or even the same client under different circumstances) might engage with TF *via* different mechanisms.

## DnaK: preferential contacts with unfolded states allow a client to find its folded structure

The heat shock protein 70 (Hsp70) family of chaperones is highly conserved and present from bacteria to humans. These chaperones likely engage in the folding of most proteins within the cell, and thus are central members of the chaperone network (12). Unlike Spy or TF, Hsp70s are ATPases that couple nucleotide state to their affinity for clients. The nucleotide-binding domain (NBD) is responsible for binding to adenosine nucleotides, and it is connected to the SBD *via* an interdomain linker (12). In the ATP-bound state, the helical lid region of the SBD is docked onto the NBD (42, 43). In contrast, the lid closes onto the rest of the SBD in the ADP state (44, 45, 46). The *Escherichia coli* Hsp70, DnaK, has been studied extensively as a representative member of this family, and structural studies have shown that large conformational changes in the SBD and NBD accompany nucleotide cycling (47). Importantly, the relative position of the lid regulates affinity for clients, as the ADP-bound, “closed” chaperone binds tightly to clients (typically low micromolar), while the ATP-bound “open” state binds comparatively weakly (typically mid-micromolar). However, DnaK has a slow intrinsic ATPase activity and therefore relies on its cochaperone DnaJ to stimulate ATP turnover and promote client refolding (12, 48). This cooperative ATPase activity has been shown to be important in unfolding activity, by which DnaK and its cochaperones can renature a misfolded protein (49).

**Figure 4:**
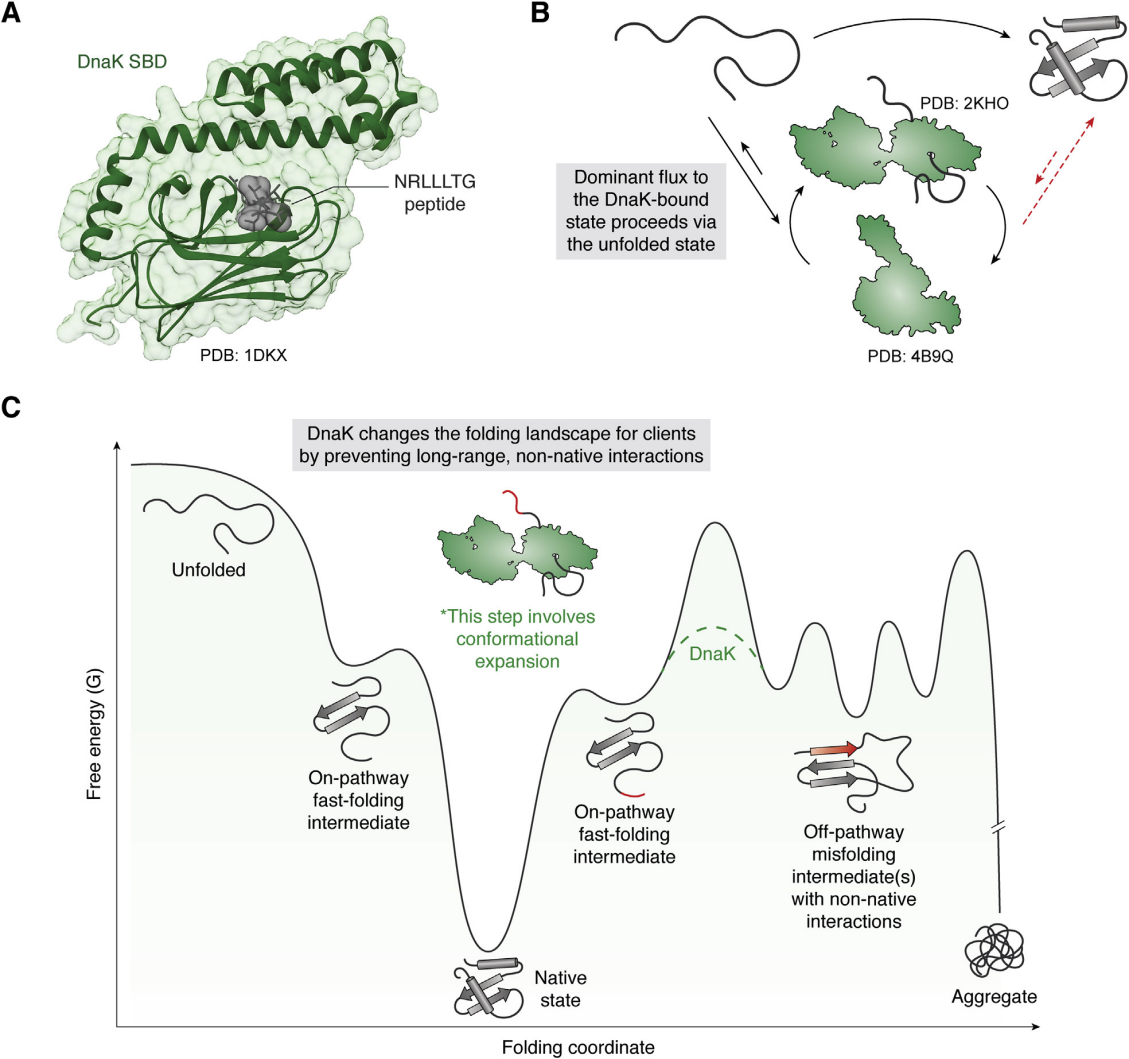
**DnaK directs clients to the native state by binding to the unfolded state and off-pathway misfolded intermediates.***A*, crystal structure of DnaK SBD bound to model NRLLLTG peptide. *B*, DnaK employs conformational selection to bind to the unfolded state, but not the native state, driving flux in the pathway toward the native state. *C*, DnaK can bind to off-pathway intermediates, preventing long-range interactions and increasing the conversion to on-pathway intermediates. DnaK is also able to intervene at multiple other points in the folding landscape, such as at the unfolded state and multiple other nonnative states. Also, there is no “directionality” implied in the action of DnaK on the topological landscape (*e.g.,* DnaK does not know: which direction leads to the native state).

How does DnaK direct its many, diverse clients along their folding trajectories? Early studies hinted at a mechanism, by showing that DnaK has a preference for hydrophobic residues in linear, extended regions of clients (50). The substrate-binding cleft in DnaK's SBD is a shallow groove that can accommodate a wide range of nonpolar residues, but it leaves little room for secondary structure (44, 45, 46) (Fig. 4*A*). Furthermore, the DnaK cochaperone DnaJ, which can guide clients to DnaK, also exhibits a strong preference for hydrophobic stretches and reinforces the specificity of DnaK (51). Together, these insights suggested that Hsp70 binds to hydrophobic stretches of ∼7–8 residues within unfolded or denatured clients. However, while these important studies identified the DnaK-binding sites in clients, they did not explain how DnaK worked to promote folding. To examine this mechanism, more information was needed about the client's structural state bound by Hsp70. As was the case in studies of Spy and TF mechanisms, modern approaches in NMR spectroscopy have proven invaluable. These studies have also taken advantage of slow-folding model clients, in which both the unfolded and folded populations can be observed. One recent example used the SH3-DnaK system to show that DnaK uses a conformational selection mechanism to preferentially bind to the unfolded client population (52). This mechanism, in which the affinity of DnaK for the unfolded state is significantly tighter than its affinity for the native state, allows the folded population to remain untouched and therefore allows DnaK to direct flux through the pathway from the unfolded to the folded state (Fig. 4*B*).

While this conformational selection mechanism ensures that DnaK does not unfold native proteins, how are clients directed toward the native state? Unlike the Spy system, clients do not fold while bound to DnaK; the substrate-binding cleft in the SBD is too narrow to accommodate secondary structure. Instead, binding of clients to DnaK changes the energy landscape of folding (Fig. 4*C*). Using a model client, human telomere repeat binding factor (hTRF1), Sekhar *et al.* (53) established that DnaK prevents long-range interactions that would normally occur between regions of the client that are on opposite sides of the DnaK binding site. Furthermore, DnaK is able to interact with extended polypeptide sequences in a number of orientations (54, 55) and at multiple, distinct sites (56), resulting in a diversity of potential starting conformations for the client upon its release and allowing it to explore a broader range on the conformational energy landscape (56). These findings have recently been supported by studies of luciferase refolding, in which DnaK binding expands a compact folding intermediate of luciferase and thus rescues kinetically trapped intermediates (Fig. 4*C*) (20). Overall, these findings suggest that, by selectively interacting with unfolded and intermediate clients, DnaK alters the folding landscape and guides flux toward the native state.

For DnaK, a key aspect of this mechanism is that ATP hydrolysis adjusts the relative affinity of the chaperone for the client. This reversibility is important because, binding of Hsp70s can be inhibitory to folding unless it is released, for example, by NEFs or Hsp90 (5, 57). It seems likely that a major role of nucleotide cycling is to catalyze final release of the chaperone. Indeed, ATP is required for DnaK-mediated client folding *in vitro*, suggesting that weak affinity alone is not sufficient, but that the cycling is essential.

## Putting it all together: folding mechanisms of three chaperones

The recent studies highlighted here allow us to ask about the similarities and differences in the mechanisms of chaperone-mediated folding by Spy, TF, and DnaK. One key similarity is that each chaperone prefers to bind the unfolded state(s) through hydrophobic contacts. Because hydrophobic residues are often confined to the interior of a native, folded state, this mechanism likely allows the chaperones to identify a potentially misfolded client by its exposed binding sites. Given the complete lack of sequence or structural homology between these three chaperones, it is striking that they all use a common, physical “logic” to discriminate folded from unfolded proteins. However, what happens after recognition of the client is somewhat different for the three chaperones. In Spy, folding occurs within the confines of the chaperone surface, after which the natively folded client is released. However, for TF and DnaK, interactions occur with extended regions, and folding does not seem to occur within the bound complex. Here, the chaperone must be released from the client before folding can proceed. TF achieves this reversibility with a carefully tuned series of affinity constants (including a critical dimer–monomer transition), while DnaK uses conformational changes powered by ATP hydrolysis. As discussed throughout, it also seems possible that the nature of the client itself, such as its hydrophobicity and domain architecture, might, in part, dictate what mechanisms it employs to fold and, potentially, which chaperones are best suited to handle it.

## HscB: coordinating final client release by coupling the folding process to cofactor installation

While the study of individual chaperones has been critical for understanding folding mechanisms, many chaperones work together to fold clients. For example, Hsp70 and Hsp90 have been demonstrated to collaborate in both bacterial and human systems (5, 58, 59, 60). In these multicomponent systems, what is the role of weak interactions in progression of client(s) toward the native state? How do the chaperones collaborate? It is easy to imagine that, because multiple chaperones often compete with each other for the same hydrophobic regions on clients, their hierarchical affinities and/or recognition of distinct client states might enforce directionality (58). However, it has proven challenging to gain structural and mechanistic insights in many multichaperone systems, especially when compared with the single chaperone examples described above. Here, we focus on the relatively well-studied group of chaperones that are involved in iron–sulfur (Fe-S) cluster biogenesis and transfer to clients. This example was selected because, as in the cases above, modern methods in NMR spectroscopy have been critical to probing the PPIs. Thus, while many of the mechanistic details are clearly unique to this specific system, a focused discussion seems likely to suggest broad lessons.

**Figure 5:**
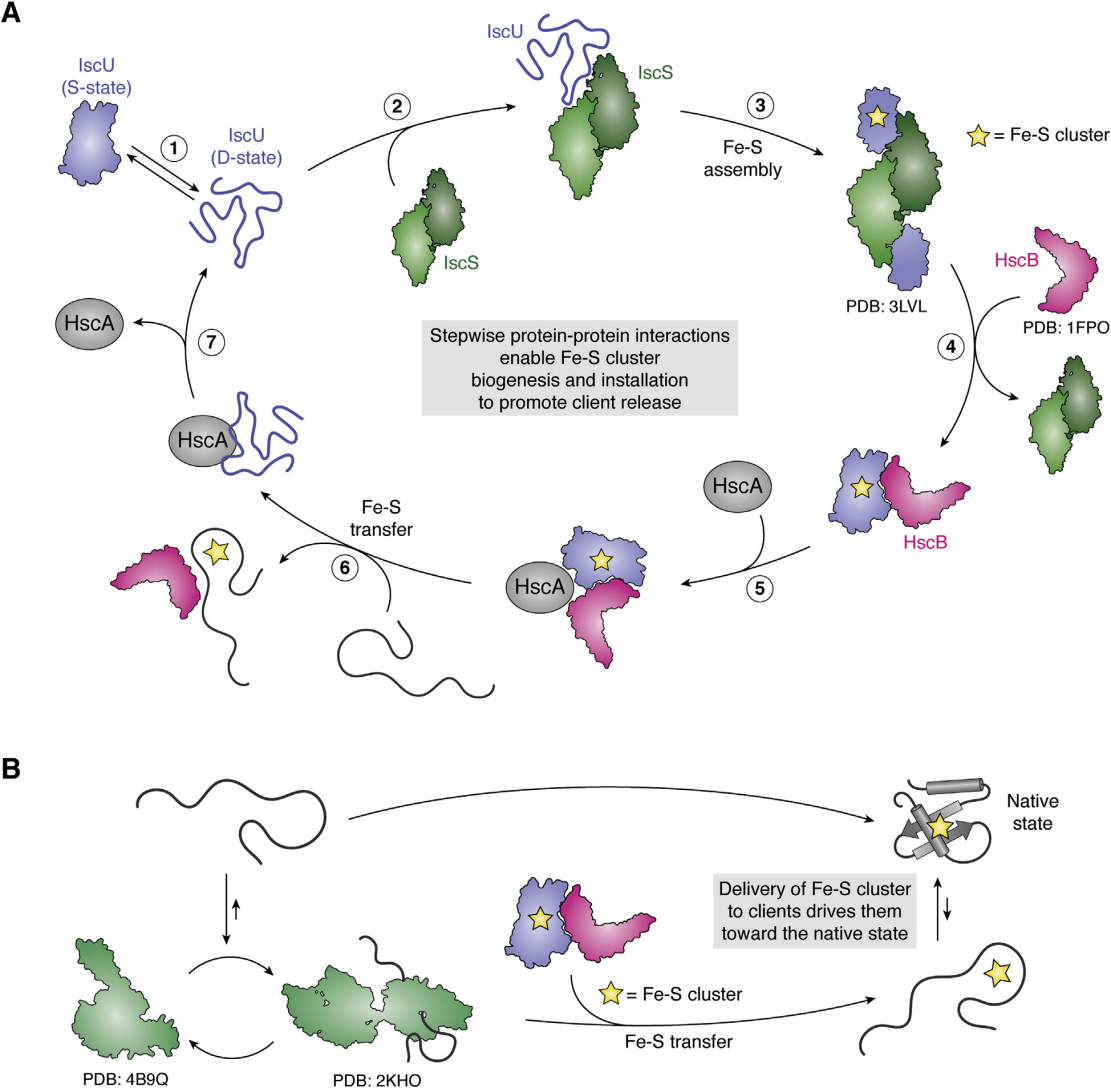
**Stepwise protein–protein interactions enable Fe-S cluster biogenesis and installation to promote client release.***A*, the cycle of Fe-S biogenesis and transfer is depicted. *1*, IscU exists in equilibrium between a structured state (S-state) and a disordered state (D-state). *2*, the cysteine desulfurase IscS interacts with the D state of IscU. *3*, assembly of the Fe-S cluster stabilizes the S state of IscU. *4*, holo-IscU is transferred from IscS to HscB. *5*, holo-IscU forms a ternary complex with HscB and HscA. *6*, Fe-S cluster is transferred to the client protein, HscB is released, and HscA binds to the D state of IscU. *7*, nucleotide exchange of HscA causes release of IscU. *B*, loading of the apo-client with the Fe-S cluster drives client release and folding.

This process can be broken down into two steps: biogenesis of Fe-S clusters, and transfer of clusters onto recipient client proteins. In bacteria, biogenesis is completed by two proteins: IscS, a cysteine desulfurase, and IscU, a scaffold protein. The second step of cluster transfer is then facilitated by two chaperones: the Hsp70 homolog HscA, and the HscA cochaperone HscB. HscB is a J-domain protein (JDP), which functions to stimulate the ATPase activity of HscA (12, 48). HscA and HscB partner with IscU to enable cluster transfer to the client. Together, these factors coordinate client folding with cluster installation in a stepwise cycle (Fig. 5*A*).

This process is driven by conformational changes associated with Fe-S cluster binding and ATP hydrolysis. Specifically, the Fe-S cluster is first formed on the scaffold protein IscU (61). Cluster formation, in turn, causes a structural change of IscU to the S-state (structured), with which HscB preferentially interacts (62, 63, 64, 65). The complex of HscB and IscU-Fe-S then interacts with HscA-ATP and the client protein, promoting ATP hydrolysis and transfer of the cluster onto the client (66, 67, 68). Following ATP hydrolysis, the cluster dissociates, and IscU converts to the D-state (disordered), which can then form new Fe-S clusters (65). The cycle is then able to repeat, with new clusters forming on IscU.

This entire cycle is driven by weak PPIs between the various components. For example, the K_d_ of apo-IscU and HscB is 9–13 μM (66, 69), 9 μM for apo-IscU and HscA-ADP (70), and 37 μM for apo-IscU and HscA-ATP (70). Additionally, there is a hierarchy to the weak PPIs: HscA shows preferential binding with the D-state of IscU, while HscB preferentially binds the S-state of IscU (65). These interactions have functional roles as well; alone, IscU and HscB stimulate HscA by 3–10 fold, but together, they synergistically stimulate ATPase activity of HscA by 500-fold (67). Furthermore, additional interactions with other proteins in the Fe-S cluster biogenesis system play a role. For example, the cysteine desulfurase IscS preferentially interacts with D-state IscU, promoting Fe-S cluster formation on IscU and interactions with HscB and HscA (64). Together, this series of weak interactions allow the system to be dynamic, forming temporary contacts that promote chaperone activity and Fe-S cluster transfer.

The Fe-S cluster system is highly conserved, with similar systems existing in eukaryotes, including yeast and humans (71, 72, 73). In these systems, it has been shown that IscU can still occupy the two different S- and D-states and has similar interactions with the JDP, Hsp70, and cysteine desulfurase (74). Importantly, a few differences do exist. While bacteria have HscA, a specialized Hsp70, dedicated to Fe-S cluster transfer, humans do not (75). Rather, the general, mitochondrial Hsp70 (mortalin, HSPA9) seems to be responsible for these functions. Additionally, bacterial and human HscB (sometimes referred to as Hsc20) are structurally similar, and both contain the conserved J-domain (71); however, human HscB contains a tetracysteine metal-binding motif, of which the exact function is not understood (76). Lastly, work by Maio *et al.* found that human HscB recognizes Fe-S cluster recipients through LYR motifs in client proteins (77, 78).

These studies show the significant role that weak PPIs have in Fe-S cluster formation and transfer. The weak PPIs allow for multiple binding partners, cycle progression and renewal, and eventual transfer of the cluster onto the appropriate recipient protein. It is the installation of the Fe-S cluster onto the client that acts as a dedicated step, causing release of the client and directing it toward the native state (Fig. 5*B*). Fe-S clusters are essential components of many enzymes, and it is therefore unsurprising that these systems are part of a tightly controlled and highly conserved cellular machinery.

Although this pathway is among the best characterized of the multicomponent chaperone systems, questions about the molecular mechanisms remain. Further work is necessary to elucidate precisely how HscB recognizes client proteins, and it is not understood what conformational changes accompany loading of Fe-S clusters into clients. Additionally, there are still gaps in our understanding of the differences between the human and bacterial systems. Nevertheless, these foundational studies establish how the weak PPIs promote directionality, even within complex chaperone systems.

## Other chaperones: similarities and many differences

To this point, we have focused on recent structural studies on Spy, TF, DnaK, and HscA-HscB to illustrate some similarities and differences in the ways that chaperones promote protein folding. As mentioned, this choice was driven by the timely convergence of recent studies, using NMR methods that provided insights into chaperone–client interactions. However, we would be remiss if we did not mention how much can be learned by comparing these findings to those from other classes of chaperones. Perhaps the best illustration of this diversity is in the GroEL/GroES chaperonin system. Briefly, GroEL forms a barrel-shaped oligomer that encompasses a central chamber. Cycles of ATP hydrolysis, PPIs with GroES, and associated conformational changes have been revealed by structural studies, showing that clients enter this cavity, where they are shielded from aberrant contacts (79, 80). In this system, client folding is likely catalyzed by iterative hydrophobic and polar interactions within the chamber. This mechanism is quite distinct from the examples mentioned above because the client is entirely isolated from bulk solvent. Still other distinct mechanisms have been revealed by recent structures of Hsp90 in complex with clients (16, 81, 82). In that system, it is becoming clear that clients are partially folded when bound to Hsp90, such that they are poised to be released by cofactor binding (perhaps akin to the Fe-S cluster example). Studies on other categories of chaperones, such as Hsp27 (83, 84, 85), clusterin (86, 87), and Hsp110 (88), have also been reported, providing an increasing number of comparative examples on which to identify patterns. However, for many chaperone classes, the molecular details are less clear. Moreover, even studies on well-known chaperones would likely benefit from exploration of additional clients, as it seems that some chaperones might be capable of accessing multiple mechanisms. For example, mechanisms such as ultra-affinity (89), entropic pulling (90, 91), and unfolding (92) have been described for Hsp70, and multichaperone systems can even disaggregate aggregated proteins (93, 94, 95, 96). As was seen in studies of Spy, different clients might access distinct binding states and, potentially, different mechanisms.

## Discussion

In this review, we have focused on recent evidence suggesting that molecular chaperones engage in weak, transient PPIs that direct clients toward the native state. These studies rely on elegant NMR-based structural work, which has revealed the mechanisms of chaperone function *in vitro*. While illuminating, it is worth noting that, in the cell, these interactions and their affinities will be further tuned by changes in the chaperone concentrations, which are dynamic and responsive to the cellular environment. For example, the concentrations of many molecular chaperones are increased when cells encounter stressful stimuli, such as high temperatures (97). In addition, chaperones (and their clients) may also be posttranslationally modified to quickly respond to changing conditions. These posttranslational modifications (PTMs) have the potential to alter the affinities of chaperones for their clients or cochaperones, as has been demonstrated for small heat shock proteins (sHsps), Hsp70, and Hsp90 (83, 98, 99, 100, 101). Continued study of such PTMs will be crucial for understanding how PPI hierarchies are dynamically regulated to make “decisions” about client fates in the cell. For example, cells may choose to stall protein folding and favor “holdase” activity under some conditions.

The weak interactions with chaperones may also play roles beyond simply directing clients to the native state. While this review has focused on chaperone-mediated folding, hierarchical interactions likely lead clients toward other fates, such as degradation. For example, some clients seem to be preferentially degraded by the ubiquitin-proteasome system when they remain bound to the Hsp70 complex for too long (102, 103), and a similar mechanism may underlie client degradation by Hsp90 complexes (104, 105). In addition to determining client fate, it is possible that weak interactions between chaperones and clients have far-reaching consequences for the evolution of client sequences and folds, because there is strong evidence that some chaperones, such as Hsp90, GroEL, and DnaK, can accelerate the sequence evolution of their clients (14, 106, 107, 108). Undoubtedly, the promiscuity and low affinity of these chaperone–client interactions allow chaperones to accommodate a changing and adaptable proteome.

Finally, one goal of understanding chaperone–client interactions is to identify potential therapeutic approaches for the treatment of protein misfolding diseases (109, 110, 111). Despite the elegant mechanisms that chaperones use to fold and degrade proteins, there are many diseases, including most neurodegenerative disorders, that are characterized by protein misfolding and aggregation. If we better understood the mechanisms of how chaperones bind and assist protein folding, maybe we could learn how to create therapies that mimic this activity? For example, Hsp70 has been shown to suppress aggregation of tau (112, 113) and synuclein (93, 94). Mimicking or promoting these activities could be beneficial in treating neurodegeneration. Chemical biology approaches have already produced chemical probes that have proven invaluable in teasing apart the molecular logic of the chaperone network (10, 114, 115, 116). We speculate that further knowledge of interaction hierarchies may unlock new ways of identifying and creating novel chemical probes that operate at the most critical of these weak PPIs. Such chemical interventions could pave the way toward next-generation therapeutics for the treatment of protein misfolding disease.

## Conflict of interest

The authors declare that they have no conflicts of interest with the contents of this article.
